# Optimization of phase contrast in bimodal amplitude modulation AFM

**DOI:** 10.3762/bjnano.6.108

**Published:** 2015-04-28

**Authors:** Mehrnoosh Damircheli, Amir F Payam, Ricardo Garcia

**Affiliations:** 1 Instituto de Ciencia de Materiales de Madrid, CSIC, Sor Juana Inés de la Cruz 3, 28049 Madrid, Spain; 2 Permanent address: Department of Mechanical Engineering, Shahr-e-Qods Branch, Islamic Azad University, Tehran, Iran

**Keywords:** bimodal AFM, dynamic AFM, tapping mode

## Abstract

Bimodal force microscopy has expanded the capabilities of atomic force microscopy (AFM) by providing high spatial resolution images, compositional contrast and quantitative mapping of material properties without compromising the data acquisition speed. In the first bimodal AFM configuration, an amplitude feedback loop keeps constant the amplitude of the first mode while the observables of the second mode have not feedback restrictions (bimodal AM). Here we study the conditions to enhance the compositional contrast in bimodal AM while imaging heterogeneous materials. The contrast has a maximum by decreasing the amplitude of the second mode. We demonstrate that the roles of the excited modes are asymmetric. The operational range of bimodal AM is maximized when the second mode is free to follow changes in the force. We also study the contrast in trimodal AFM by analyzing the kinetic energy ratios. The phase contrast improves by decreasing the energy of second mode relative to those of the first and third modes.

## Introduction

The atomic force microscope is a versatile and powerful tool for imaging, compositional mapping and modification of surfaces with atomic and nanoscale spatial resolutions [1–8]. The evolution of AFM is being shaped by the need to provide images of heterogeneous surfaces with high spatial resolution combined with compositional contrast and/or material properties mapping [7,9]. Amplitude modulation force microscopy (AM-AFM) was designed to excite the cantilever near or at its fundamental free resonant frequency [2]. However, the need to improve and/or provide quantitative compositional contrast without compromising the data acquisition speed has led to the development of several AFM modes, specifically multifrequency force microscopy methods [9–32].

**Figure 1:**
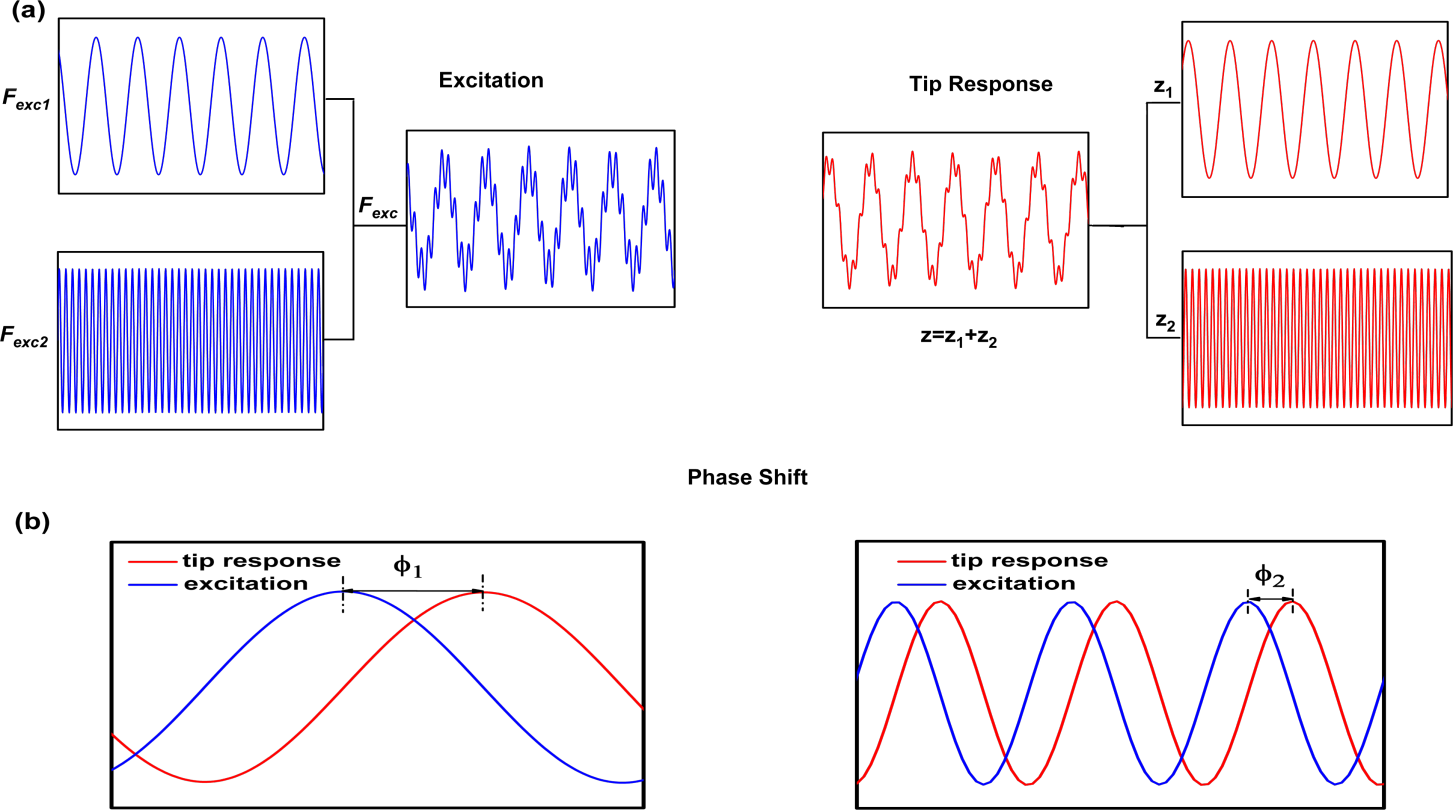
(a) Scheme of the excitation and detection signals in bimodal AM configuration. (b) Definition of phase shifts in bimodal AM for the first and the second modes.

Bimodal force microscopy is a multifrequency AFM method that uses two eigenmode frequencies for excitation and detection (Figure 1) [9]. It has several configurations depending on the feedback schemes [16–24]. In the first bimodal AFM configuration (bimodal AM) [15–16], the feedback acts on the amplitude of the first mode by keeping it at a fixed value during imaging while the second mode operates in an open loop. The ability of bimodal AM to map compositional variations under the influence of conservative forces is a main advantage with respect to AFM phase imaging (tapping mode AFM), where the phase contrast is related to variations in energy dissipation [33].

In AM-AFM there are two interacting regimes, attractive and repulsive [2]. The regimes are defined by the average value of the force [34]. A transition between the regimes is usually accompanied by a sudden change of the observables (amplitude and phase shift). In bimodal AFM some additional contrast regimes has been identified [35–37] where sudden changes of the phase contrast are not associated with changes in the sign of the average value of the force. The origin of those regimes are discussed in terms of the different energies of the system, kinetic energy of the exited modes [35–38], the input energy [36] or the energy transfer between the modes [37]. In general those regimes appear when the modes are highly coupled. This happens when the energy of the first and second mode are comparable [35].

This context has also stimulated other multifrequency AFM variations such as trimodal AFM [39–41]. In trimodal AFM the first three flexural modes are excited and detected. The feedback operates on the amplitude of the first mode while both second and third modes are in open loops. It has been shown the usefulness of the third mode to modulate the indentation [23]. A comparison of the trade-offs in sensitivity and sample depth have been performed with bimodal and trimodal AFM in the repulsive regime [41]. However, a similar comparison has not been reported for the attractive interaction regime.

In bimodal AFM (Figure 1), the advances in instrumentation are ahead of its theoretical understanding. To bridge the gap between experiments and theory we study the conditions to optimize the compositional contrast and material properties sensitivity in bimodal AM. The compositional contrast is usually defined as the phase shift difference of the second mode between two regions of the surface of a heterogeneous material. We study the phase contrast as a function of the amplitude ratio, the amplitude values of the second mode and the kinetic energy ratios of the excited modes. We also study the phase contrast between different materials by including energy dissipation in the tip–sample interaction, by inverting the roles of the excited modes (indirect bimodal AM) as well as in trimodal AFM. In the latter, the phase contrast is maximized when the energy of the second mode is much smaller than the other excited modes.

## Results and Discussion

### Equation of motion and tip–surface forces

To analyze the dependence of the phase contrast on the values of the different parameters we have used numerical simulations. For this we consider that bimodal AFM is characterized by the simultaneous excitation of two cantilever resonant frequencies, usually the lowest flexural eigenmodes [42]. The total driving force is expressed as

[1]
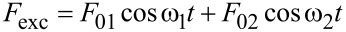


Then, the cantilever–tip ensemble will be described by the system of two differential modal equations,

[2]
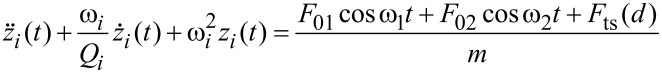


[3]
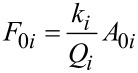


[4]
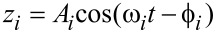


with *i =* 1,2; ω*_i_*, *k_i_*, *Q_i_*, 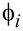
*, A_i_* and *A*_0_*_i_* are, respectively, the angular frequency, the force constant, quality factor, phase shift, amplitude and free amplitude of mode *i*; *m* = 0.25*m*_c_ is an effective mass while *m*_c_ is the real cantilever–tip mass. The solution of the above equation has two components *z*_1_ and *z*_2_ that vibrate, respectively, with the eigenmode frequencies ω_1_ and ω_2_. The instantaneous tip–surface distance *d* is defined by

[5]
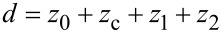


where *z*_0_ and *z*_c_ are respectively, the average tip deflection and the average tip–surface separation. The tip–surface force is modelled by Equation 6 where *a*_0_ is a molecular distance (0.165 nm).

[6]
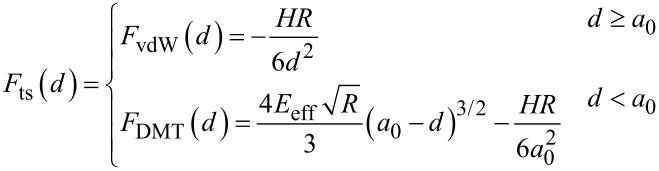


### Material and cantilever–tip parameters

To study the phase contrast we have simulated the bimodal AM operation for two materials gold (Au) and polystyrene (PS). The values of the material properties needed to describe the tip–surface force (Hamaker constant and Young modulus) and the operational values of the microscope are summarized in Table 1 and Table 2.

**Table 1:** Hamaker and Young modulus values.

*H* (tip-gold)	*E* (Au)	*H* (tip-PS)	*E* (PS)

12 × 10^−20^ J	75 GPa	7 × 10^−20^ J	3 GPa

**Table 2:** Cantilever-tip parameters.

*R* (nm)	*k*_1_ (N/m)	*f*_1_ (kHz)	*Q*_1_	*k*_2_ (N/m)	*f*_2_ (kHz)	*Q*_2_

20	0.896	49.16	254	35.24	308.26	995

For trimodal AFM simulations we have used the parameters shown in Table 3. To minimize some complex non-linear dynamic effects we restrict our study to situations that involve the attractive regime. The attractive forces have been modeled by van der Waals interactions with the Hamaker values given in Table 1.

**Table 3:** Cantilever–tip parameters in trimodal AFM.

*R* (nm)	*k*_1_ (N/m)	*f*_1_ (kHz)	*Q*_1_	*k*_2_ (N/m)	*f*_2_ (kHz)	*Q*_2_	*k*_3_ (N/m)	*f*_3_ (kHz)	*Q*_3_

20	0.896	49.16	254	35.24	308.26	995	276.18	862.86	766

### Phase contrast in the attractive regime (conservative force): *A*_01_ > *A*_02_

In bimodal AM the feedback loop operates on *A*_1_, consequently the amplitude of the first mode or its ratio is the relevant parameter to be used as the independent variable. In some cases, the representation with respect to the average tip–surface distance could also provide useful information.

**Figure 2:**
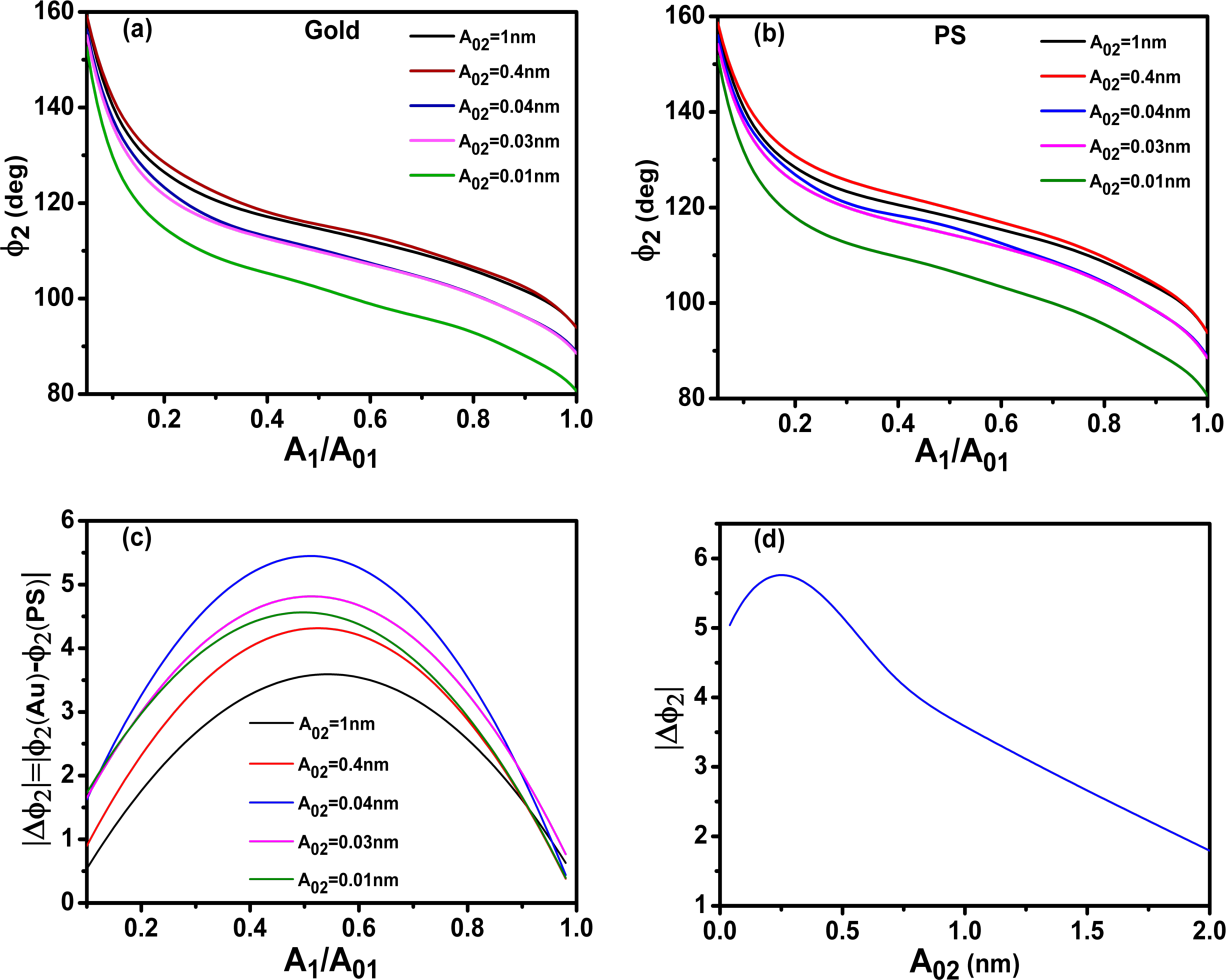
Bimodal AM in the attractive regime. (a) Phase shift dependence on the amplitude ratio of the first mode for different values of *A*_02_. The value of the Hamaker constant is set for the Au–air–Si interface. (b) Phase shift dependence on the amplitude ratio of the first mode for different values of *A*_02_. The value of the Hamaker constant is set for the PS–air–Si interface. (c) Phase contrast between Au and PS as a function of the amplitude ratio. (d) Phase contrast as a function of *A*_02_. *A*_01_ = 10 nm; see Table 1 and Table 2 for more details.

The dependence of 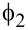 with *A*_1_/*A*_01_ has been described previously [15–16]. In the attractive regime, it shows an increase with *A*_1_/*A*_01_ decreasing (*A*_01_ = 10 nm). The fastest changes happen at the edges of the *x*-axis (small and large amplitude ratios). This behavior is reproduced for gold (Figure 2a) and PS (Figure 2b) for different values of *A*_02_. Interestingly, for the same *z*_c_ the phase shift is larger for the material with the smaller Hamaker value. This is at odds what happens in amplitude modulation AFM, where the phase shifts increases with the value of *H*.

The phase contrast │Δ
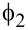
│=│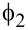 (gold) − 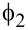 (PS)│ depends on both the *A*_1_/*A*_01_-ratio and the value of *A*_02_. Two maxima are observed, one with respect to *A*_1_/*A*_01_ and the other with respect to *A*_02_. The first maximum happens near an *A*_1_/*A*_01_-ratio of about 0.5. It seems similar to the behavior observed in AM-AFM for the dependence of the minimum distance with *A*_1_/*A*_01_ [43]. In terms of optimizing the material contrast it is more relevant to pay attention to the behavior with respect to *A*_02_ (Figure 2d). It shows the phase contrast for *A*_01_/*A*_02_ ratios between 5 and 2000. Small values of *A*_02_ are needed to enhance the material contrast, however, for very small *A*_02_ the bimodal enhancement of contrast will disappear as the system becomes monomodal, i.e., tapping mode AFM. For this simulation the best contrast is yielded for an amplitude ratio of 250. This value is significantly larger than the values previously recommended (10–50) which were based on experiments [43–45].

### Phase contrast in the attractive regime (dissipation): *A*_01_ > *A*_02_

To study the effect of energy dissipation in the bimodal phase contrast, in addition the above conservative force, we introduce the following non-conservative interaction [47]:

[7]
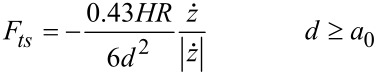


The power dissipated in the sample for each mode is calculated by [47]

[8]
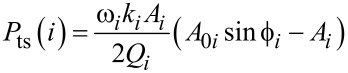


**Figure 3:**
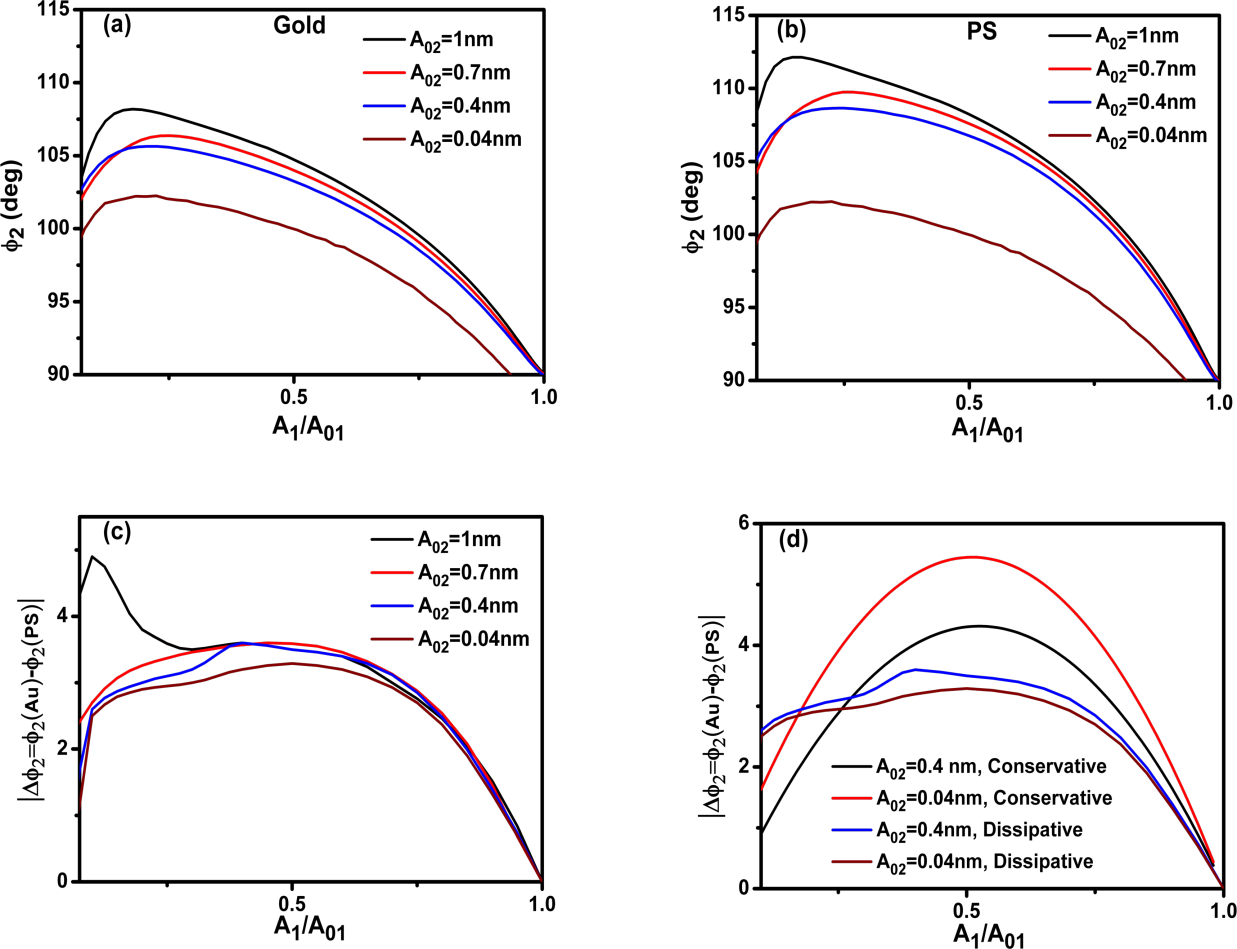
Phase contrast in bimodal AM in the presence of dissipation (attractive regime). (a) Phase shift dependence on the amplitude ratio of the first mode for different values of *A*_02_. The value of the Hamaker constant is set for the Au–air–Si interface. (b) Phase shift dependence on the amplitude ratio of the first mode for different values of *A*_02_. The value of the Hamaker constant is set for the PS–air–Si interface. (c) Phase contrast between Au and PS as a function of the amplitude ratio. (d) Comparison of the phase contrast between Au and PS with and without dissipation. *A*_01_ = 10 nm.

Figure 3a,b show the dependence of 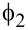 versus *A*_1_/*A*_01_ when the tip–sample interaction includes non-conservative interactions. The phase shift increases by reducing the *A*_1_/*A*_01_-ratio until a maximum is reached for ratios below 0.2. This behavior is reproduced for both gold and PS and for different *A*_02_ values. The increase of the phase shift by increasing the value of *A*_02_ is in agreement with experimental observations [36]. The presence of dissipation reduces the phase shift for the same *A*_1_/*A*_01_-ratio (see Figure 2). Energy dissipation in the sample softens the resonance curves which in turns reduces the phase shift. This is a common feature of resonators that is not affected by bimodal excitation. The phase contrast │Δ
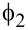
│=│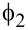 (gold) − 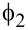 (PS)│ also shows a maximum with respect to *A*_1_/*A*_01_. The behavior is very similar to the one observed for conservative interactions (Figure 2c) except for *A*_02_ ≥ 1 nm where the maximum is displaced to very small amplitude ratios. This is due to the cross-over in the amount of power dissipated between Au and PS for those amplitude ratios (see below). In general, the introduction of dissipation processes in the tip–sample interaction reduces the material contrast observed in the phase shift of the 2nd mode (Figure 3d). This is in contrast with phase imaging in amplitude modulation AFM, where the contrast is related to energy dissipation processes. It shows that the phase contrast in bimodal AM is dominated by conservative forces [42,47]. The presence of dissipation also modifies the conditions to maximize the phase contrast to smaller *A*_01_/*A*_02_ values (20 versus 250 (no dissipation)).

**Figure 4:**
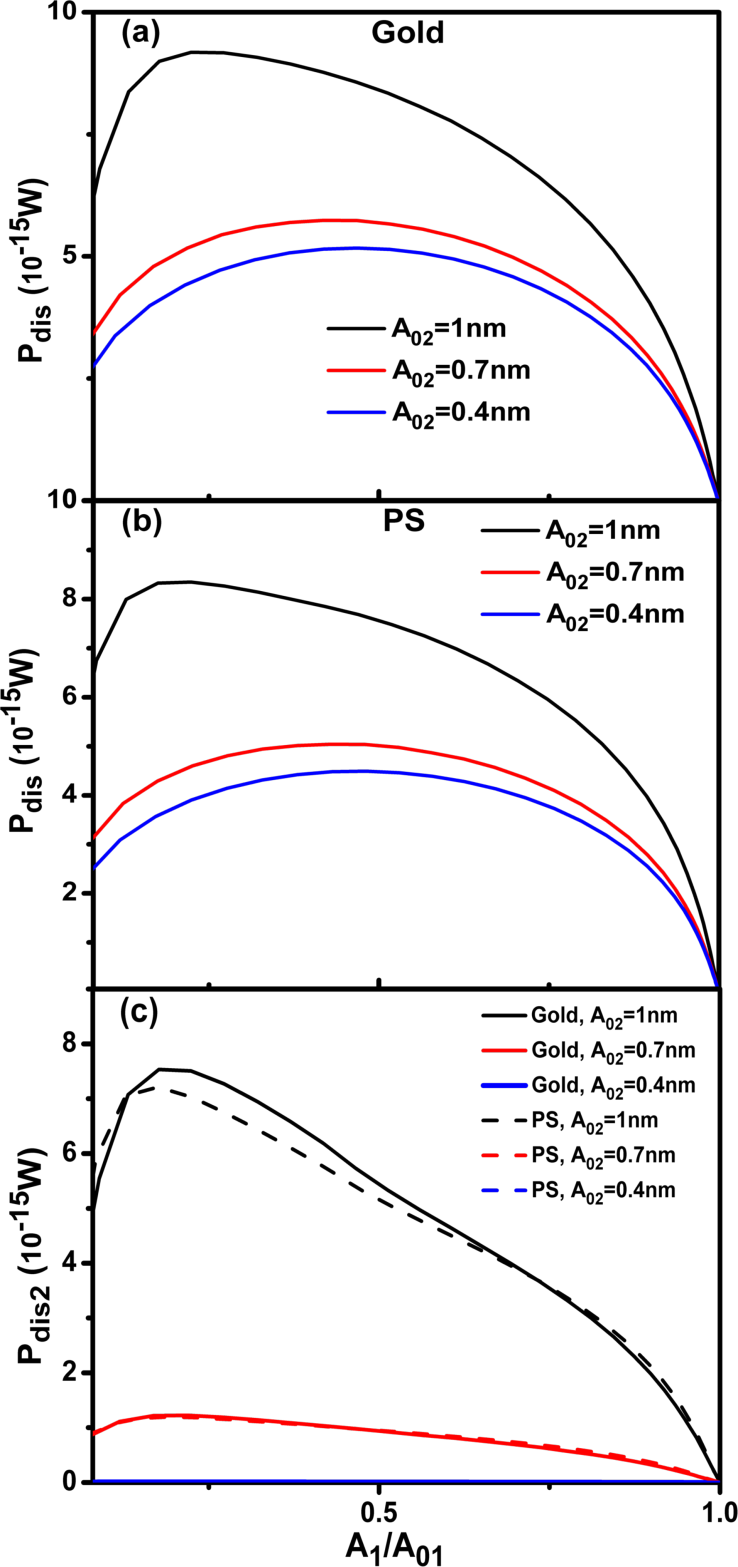
(a) Total dissipated power as a function of *A*_1_/*A*_01_ (gold). (b) Total dissipated power as a function of *A*_1_/*A*_01_ (PS). (c) Power dissipated by the second mode for different values of *A*_02_. *A*_01_= 10 nm. See Table 1 and Table 2 for more details.

To clarify the dependence of the phase contrast with the power dissipated by the tip–sample interaction we plot the dissipated power as a function of *A*_1_/*A*_01_ for different materials. Figure 4a and 4b show, respectively, the total dissipated power for Au and PS_._ The dissipated power increases with the free amplitude of the 2nd mode and it has a maximum with respect to *A*_1_/*A*_01_. This maximum is related to the existence of a minimum in the closest tip–surface separation as a function of *A*_1_/*A*_01_. More dissipation is obtained for gold than PS because the Au–air–Si interface has a higher Hamaker constant. The power dissipated by the 2nd mode also shows a maximum with *A*_1_/*A*_01_ near 0.2 (Figure 4c). A discussion about the energy transfer among different modes is presented by Solares and co-workers [48]. The data plotted in Figure 3 and Figure 4 has been obtained by using the dForce simulator [49].

### Phase contrast in the attractive regime (no dissipation): *A*_02_ > *A*_01_ (inverted bimodal excitation)

In the first bimodal AM experiments the first mode carried the feedback controls while the second has an open loop (no feedback). This configuration introduced a significant asymmetry between the roles of the excited modes. This raises the question about the equivalence of the excited modes 1 and 2 for bimodal AM operation. To answer this question we have simulated a situation where the feedback operates in the second mode while the first mode has an open loop (inverted bimodal excitation). In the simulations, the free amplitude of the second mode is 10 nm while the one of the first ranges between 0.7 and 2 nm. Other relevant parameters are described in Tables 1, 2 and 3. The cross-mode representation has 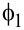 or Δ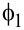 as dependent variables with respect to *A*_2_ or its ratio.

**Figure 5:**
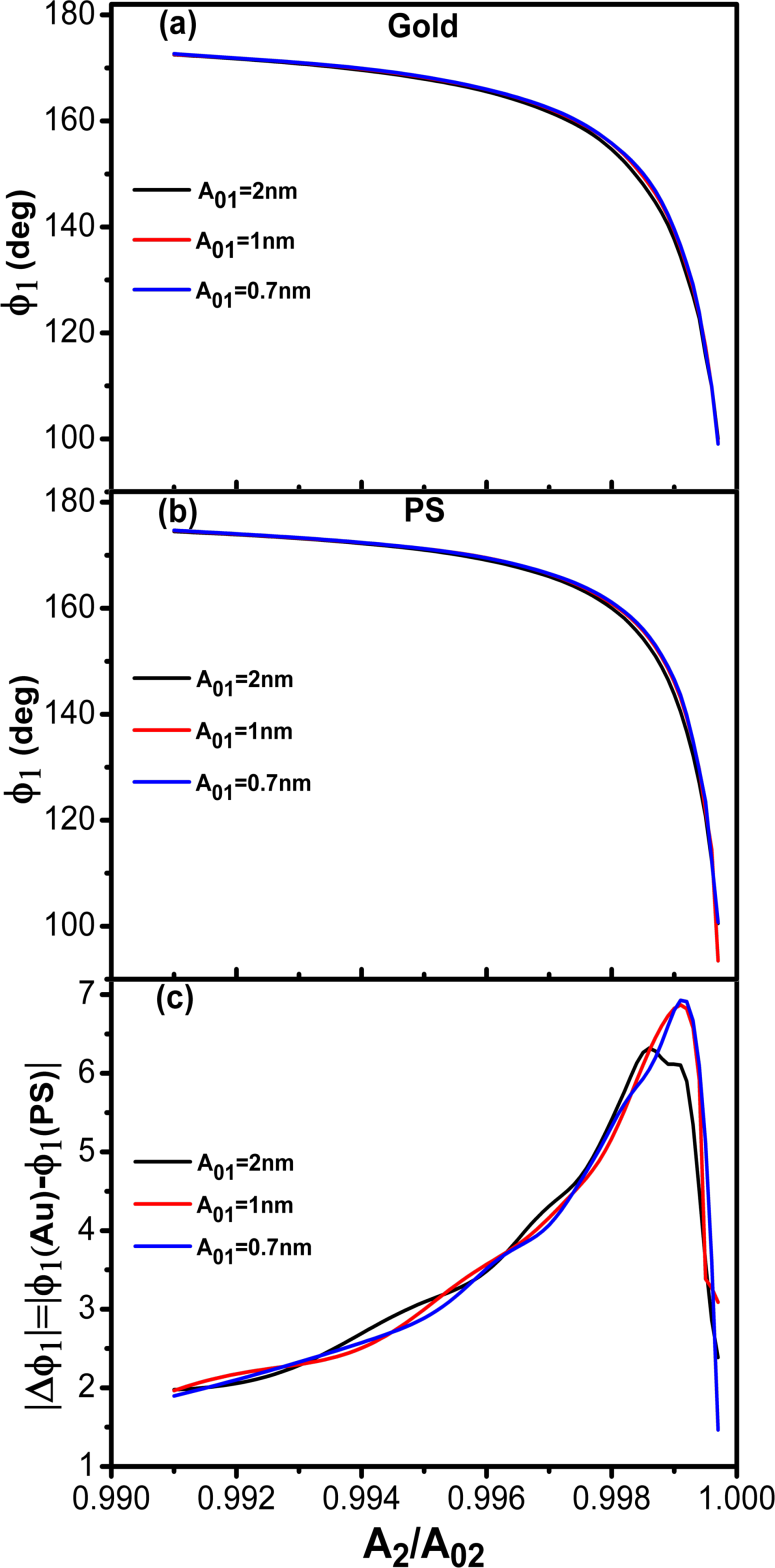
Inverted bimodal AM. (a) Phase shift dependence on the amplitude ratio (*A*_2_/*A*_02_). The value of the Hamaker constant is set for the Au–air–Si interface. (b) Phase shift dependence on the amplitude ratio (*A*_2_/*A*_02_). The value of the Hamaker constant is set for the PS–air–Si interface. (c) Phase contrast as a function of *A*_2_/*A*_02_. *A*_02_ = 10 nm; see Table 1 and Table 2 for more details.

The phase shift 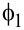 versus *A*_2_/*A*_02_ shows a quick increase from 90° to close to 180° for a rather small reduction of the amplitude ratio (Figure 5a,b). In the inverted bimodal AM there is the phase contrast between Au and PS. In fact the contrast in terms of degrees is comparable to the one observed in bimodal AM, however, it happens for an extremely small range of set-point amplitudes ratios (0.99 and 0.999). This makes it impractical from the experimental point of view. In the direct bimodal AM the phase contrast is observed in almost all the amplitude ratio range from 0.1 to 0.99.

**Figure 6:**
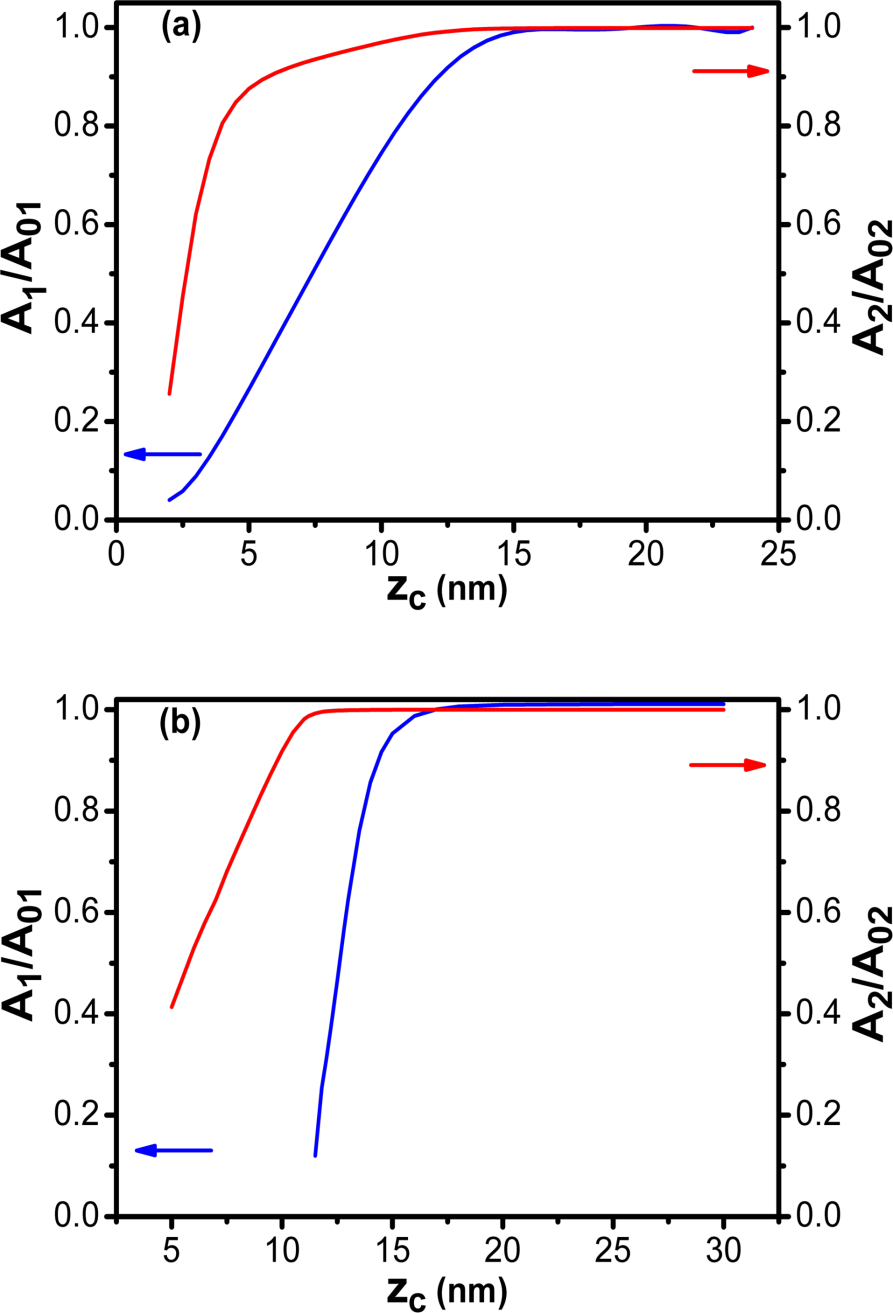
Comparison between direct and inverted bimodal AM. (a) Amplitude ratio dependence on the average tip–surface separation for bimodal AM (feedback on *A*_1_). (b) Amplitude ratio dependence on the average tip–surface separation for the inverted bimodal AM (feedback on *A*_2_).

Consequently, the roles of modes 1 and 2 in bimodal AM are not equivalent. The asymmetry is more clearly seen by plotting the dependences of *A*_1_/*A*_01_ and *A*_2_/*A*_02_ with respect to *z*_c_ for both the direct and the inverse bimodal AFM configurations (Figure 6). In the direct bimodal AM both *A*_1_ and *A*_2_ decrease with respect *z*_c_ over similar range (Figure 6a). However, in inverted bimodal AM, *A*_1_ has almost vanished while *A*_2_ is still starting to notice the presence of the tip–surface force (Figure 6b). The origin of this asymmetry can be traced back to the sensitivity of an oscillating system with respect to *Q_i_* and *k_i_*. It has been shown that the phase shift sensitivity is proportional to the *Q_i_*/*k_i_* ratio [43]. This ratio decreases with increasing the eigenmode index [9].

### Phase contrast in the repulsive regime (no dissipation): *A*_01_ > *A*_02_

**Figure 7:**
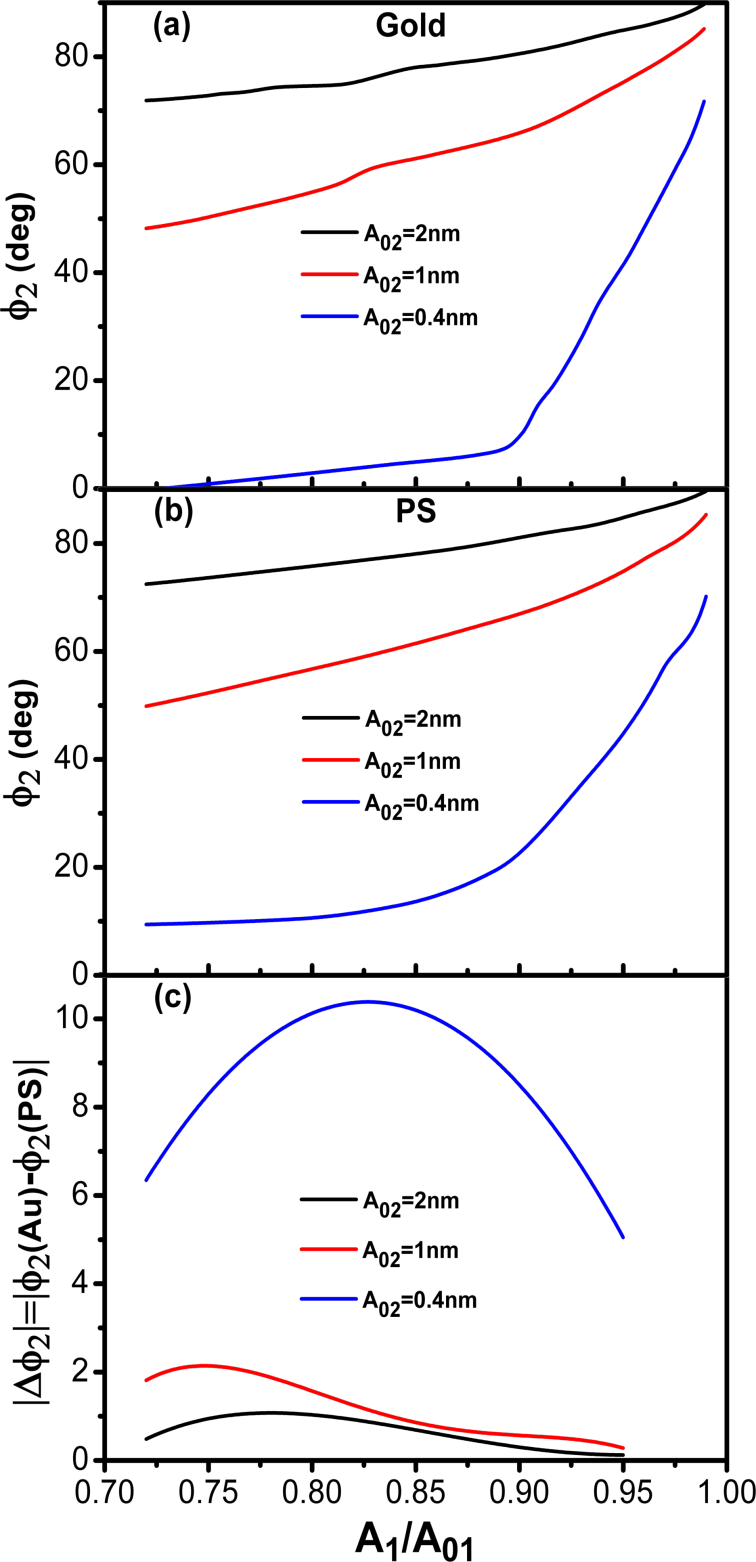
Bimodal AM in the repulsive regime. (a) Phase shift dependence on the amplitude ratio of the first mode for different values of *A*_02_. The value of the Young modulus corresponds to Au. (b) Phase shift dependence on the amplitude ratio of the first mode for different values of *A*_02_. The value of the Young modulus corresponds to PS. (c) Phase contrast between Au and PS as a function of the amplitude ratio for different *A*_02_. *A*_01_ = 10 nm, see Table 1 and Table 2 for other parameters.

In the repulsive regime, the phase shift decreases from the non-interacting phase shift (90°) with *A*_1_/*A*_01_ decreasing (Figure 7a and 7b). The decrease depends on the Young modulus and on the value of *A*_02_. For the same *A*_1_/*A*_01_-ratio lower phase shift values are observed on the stiffer material. The dependence on *A*_02_ follows the trend observed in the attractive regime. For the same *A*_1_/*A*_01_-ratio by reducing the value from 2 to 0.4 nm the phase shift variation (from the non-interacting value, 90°) is increased. More significantly, the phase contrast measured between Au and PS is also enhanced by reducing *A*_02_. A maximum is observed in the phase contrast dependence on the *A*_1_/*A*_01_-ratio (Figure 7c). The position of the maximum depends on *A*_02_. The lower the value of *A*_02_, the higher the *A*_1_/*A*_01_-ratio where the maximum is observed.

### Trimodal AFM in the attractive regime

Solares and co-workers have extended the bimodal scheme by introducing an additional excitation in the third mode [39–41,46]. The third excitation in trimodal AFM offers two additional channels for compositional contrast. The value of *A*_03_ has been used modulate the indentation while imaging embedded nanoparticles in a soft polymer [23]. To understand some of the fundamental aspects of trimodal AFM and the differences with respect to bimodal AM we study the phase contrast in trimodal AFM in the attractive regime.

We have performed simulations by using an excitation force with contributions to the first three eigenmodes

[9]
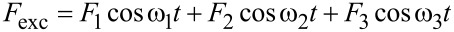


The phase contrast is studied in terms of the kinetic energy (KE) of the excited modes [38]. It has been shown that the contrast reversal observed in bimodal AM depends on the relative kinetic energy maxima of the excited modes [35–36].

[10]
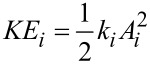


The kinetic energy analysis is applied to establish the optimum conditions for phase contrast in trimodal AFM. Table 4 shows the different amplitudes values used in the simulations and the corresponding kinetic energy relationships.

**Table 4:** Kinetic energy maxima and free amplitudes in trimodal AFM.

kinetic energy relationship	*A*_01_ (nm)	*A*_02_ (nm)	*A*_03_ (nm)

*KE*_1_ = *KE*_2_ = *KE*_3_	10	1.6	0.57
*KE*_1_ > *KE*_2_; *KE*_2_ < *KE*_3_	10	0.4	0.4
*KE*_1_ > *KE*_2_ > *KE*_3_	10	1.2	0.3

**Figure 8:**
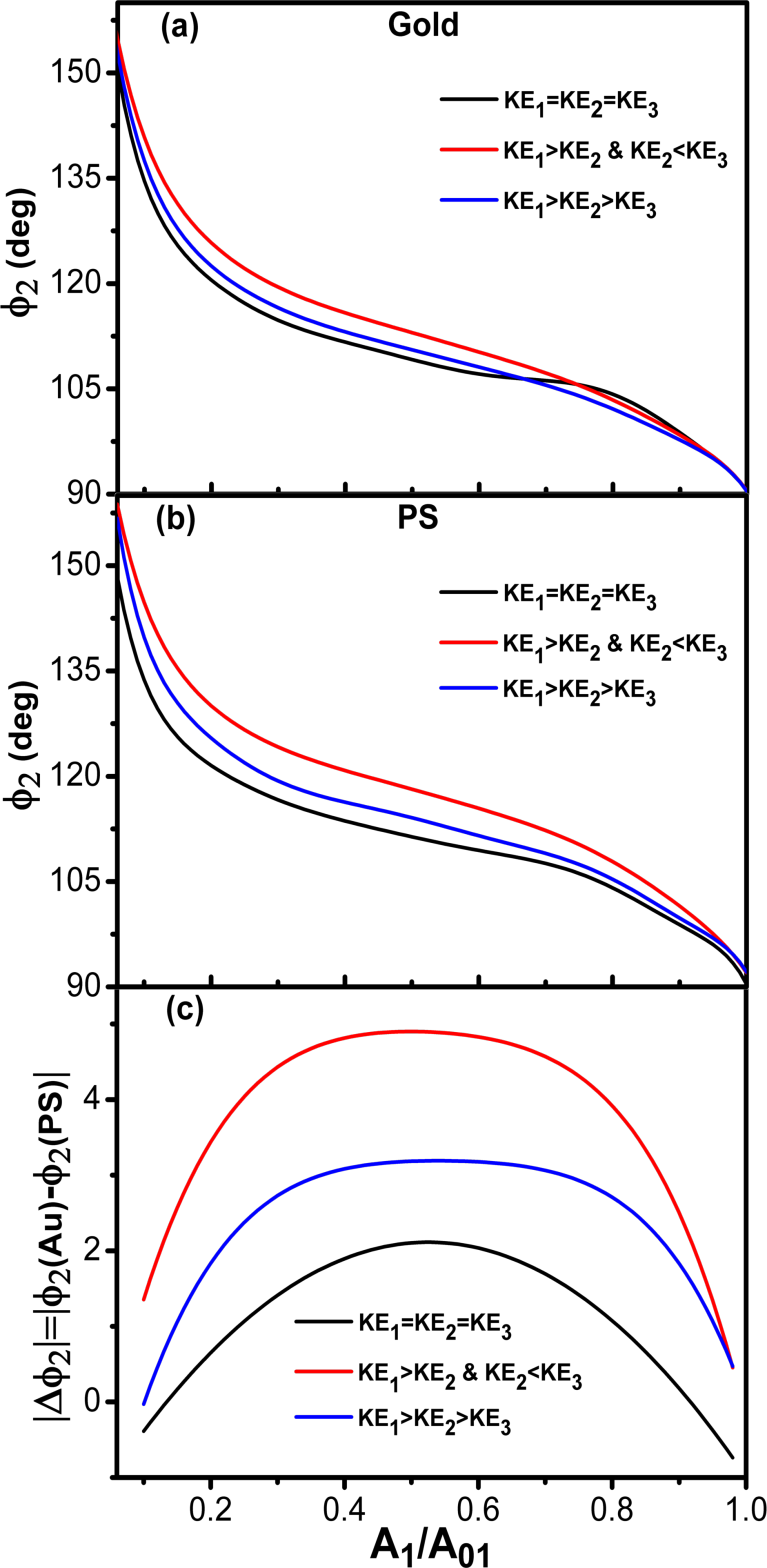
Trimodal AFM in the attractive regime. (a) Phase shift of the second mode dependence on *A*_1_/*A*_01_ for different kinetic energy ratios. The value of the Hamaker constant corresponds to Au–air–Si interface. (b) Phase shift of the second mode dependence on *A*_1_/*A*_01_ for different kinetic energy ratios. The value of the Hamaker constant is for the PS–air–Si interface. (c) Phase contrast between Au and PS as a function of the amplitude ratio for different kinetic energy ratios. See Table 3 for parameter values.

Figure 8a,b show the phase shift as function of the set-point amplitude for different energy ratios among modes. Each single curve reproduces the bimodal AM shape described before (Figure 2). Phase contrast between AU and PS is observed in all the cases irrespective of the kinetic energy distribution among the excited modes. However, the maximum contrast is obtained for a situation that minimizes the kinetic energy of the second mode with respect to the other two (Figure 8c). We also observe that the maximum contrast happens for an amplitude ratio about 0.5. This is far from the edge regions where the phase shift changes more rapidly.

**Figure 9:**
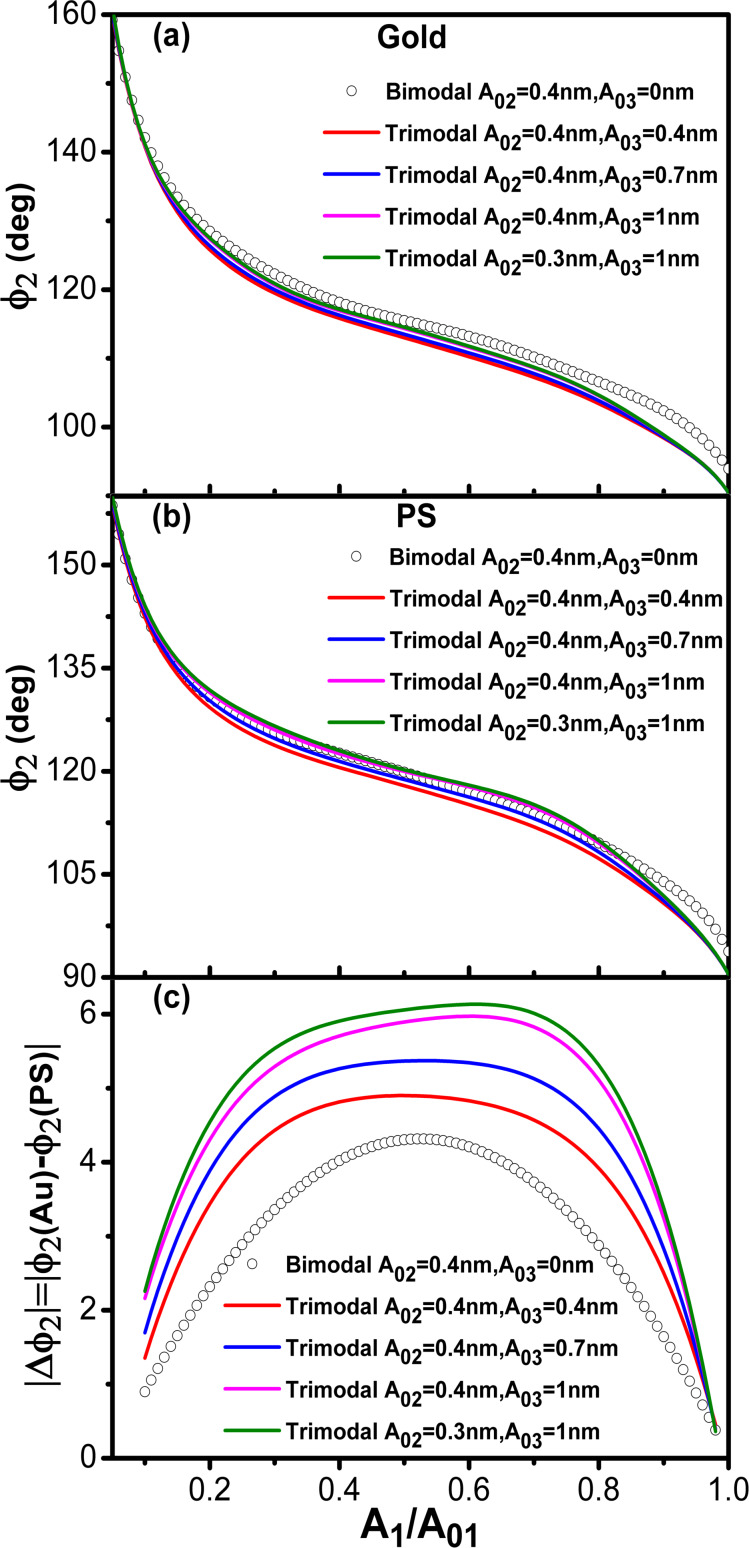
Bimodal versus trimodal AM (attractive regime). (a) Phase shift of the second mode dependence on *A*_1_/*A*_01_ for different *A*_03_ values. The value of the Hamaker constant is for the Au–air–Si interface. (b) Phase shift of the second mode dependence on *A*_1_/*A*_01_ for different *A*_03_ values. The value of the Hamaker constant is for the PS–air–Si interface. (c) Phase contrast between Au and PS as a function of the amplitude ratio for different kinetic energy ratios. *A*_01_ = 10 nm in all cases.

We have also compared the phase contrast between bimodal and trimodal AFM (attractive regime). The shape of the phase shift curves are almost identical in bimodal and trimodal AM (Figure 9a,b). However, the introduction of third excitation improves the phase contrast (Figure 9c). This seems an advantage of trimodal with respect to bimodal AM, however, this happens at the expense of introducing additional electronic hardware and increasing the peak force. A more detailed study is needed to stablish the advantages/disadvantages of these multifrequency AFM configurations.

## Conclusion

We have studied the phase contrast in bimodal amplitude modulation AFM for the attractive and the repulsive interaction regimes as a function of the amplitude and amplitude ratio of the excited modes. We have found that the contrast increases by minimizing the amplitude of the second mode. We have also compared the phase contrast for direct (conventional) and indirect bimodal AM configurations. We have found that bimodal AM favors the use of feedback controls on the amplitude of the lowest excited mode. This excitation/detection scheme maximizes the operational range. In the inverted bimodal AM configuration, the amplitude of the lowest excited mode disappears so quickly that only a very small range of amplitude ratios is left to perform bimodal AM. The origin of this asymmetry lies in the fact that the cantilever sensitivity to forces decreases with the mode number. The presence of tip–sample energy dissipation processes reduces the phase contrast observed between different materials in bimodal AM with respect to have exclusively non-conservative interactions.

The simulations show that in the attractive regime, the introduction of a small excitation in the third flexural mode improves the phase contrast with respect to bimodal AFM. This result is related to the distribution of the kinetic energies among the modes. In terms of compositional contrast it is favored the configuration that minimizes the kinetic energy of the imaging mode (second) with respect to any of the kinetic energies of the other modes. However, the increase in compositional sensitivity happens at the expense of increasing the peak forces.
